# Association between loneliness and hippocampal responses to dynamic social stimuli in psychotic disorders

**DOI:** 10.1017/S003329172510250X

**Published:** 2025-12-29

**Authors:** Faye McKenna, Louis N. Vinke, Francesca de Marneffe, Mona Avananki, Daphne J. Holt

**Affiliations:** Department of Psychiatry and Athinoula A. Martinos Center, Massachusetts General Hospital, Boston, MA, USA

**Keywords:** basal ganglia, functional MRI, hippocampus, isolation, loneliness, psychotic disorders, trust

## Abstract

**Background:**

Rates of loneliness have increased over the past several decades worldwide, particularly among people with serious mental illnesses. A better understanding of the neurocognitive mechanisms underlying loneliness could provide useful information for the efforts to address this public health problem.

**Methods:**

To investigate these mechanisms, a functional magnetic resonance imaging (fMRI) study was conducted which accounted for known cognitive biases associated with loneliness. Participants with (*n* = 40) and without (*n* = 60) psychotic disorders (PD) viewed images of faces that appeared to approach or withdraw from the participants while fMRI data were collected. Following the scanning, participants rated the trustworthiness of the faces, and these ratings were included as weights in the fMRI analyses. Neural responses to approaching versus withdrawing faces were measured, and whole-brain regression analyses, with loneliness as the regressor, were performed.

**Results:**

In the PD and full samples, a higher level of loneliness was significantly associated with greater responses of the hippocampus and areas of the basal ganglia to withdrawing (versus approaching) face stimuli. Moreover, the effects in the hippocampus, but not the basal ganglia, remained significant after controlling for potential confounds such as social activity levels, depression and social anhedonia. Finally, in a subset of the full sample (*n* = 66), greater hippocampal responses to withdrawing faces predicted greater loneliness 1 year later.

**Conclusions:**

Heightened responses of the hippocampus to withdrawing faces may represent a candidate neurobiological marker of loneliness that could be modified by interventions targeting loneliness.

## Introduction

Loneliness has been defined as a perceived deficit in social connection (a discrepancy between desired and actual levels of social activity or bonds) which can be distinguished from social isolation, an objective deficit in social activity (Cacioppo et al., 2006). In recent years, public health experts have called attention to a worsening **‘**epidemic’ of loneliness and social isolation in the general population that has been ongoing for decades (Banerjee & Rai, 2020; Killgore, Cloonan, Taylor, & Dailey, 2020). In addition, it has been well-established that loneliness and isolation are common among those suffering from psychiatric illnesses, including psychotic disorders, affecting at least 80% of people with schizophrenia (Badcock et al., 2019; Ku, Compton, Walker, & Druss, 2022; Stain et al., 2012). These findings initially seemed to contradict some longstanding assumptions about people with schizophrenia – that they do not experience the distress (and thus loneliness) often associated with social isolation. However, it has been established that emotional responses and internal affective experiences in individuals with psychotic disorders are similar to those without these illnesses (Kring, Kerr, Smith, & Neale, 1993; Moran, Culbreth, & Barch, 2022). The widespread societal problem of loneliness has broad public health implications, since numerous epidemiological studies have linked loneliness and isolation to poor cardiometabolic health and earlier mortality, health issues that have been well-documented to affect the majority of people with schizophrenia (Holt-Lunstad, Smith, Baker, Harris, & Stephenson, 2015; Holt, 2025; Correll et al., 2022). However, little is known about the psychological and neurobiological underpinnings of the associations between loneliness and poor health outcomes and how they might vary across different populations.

Some clues about the psychological mechanisms of loneliness have emerged from studies showing that certain cognitive and behavioral biases are associated with loneliness, including a bias toward mistrust of other people (Garcia-Leon et al., 2021; Lieberz et al., 2021) and a potentially related tendency to prefer a greater amount of physical space (‘personal space’) from others (Layden, Cacioppo, & Cacioppo, 2018; Saporta et al., 2021). Specifically, loneliness has been linked to a sensitivity to social rejection and a mistrust of others’ intentions (Lieberz et al., 2021), which can paradoxically lead to social withdrawal or avoidance of social activity (and thus greater isolation and loneliness). Since individuals with psychotic disorders commonly experience mistrust (even in the absence of frank persecutory delusions) (Murphy, Bentall, Freeman, O’Rourke, & Hutton, 2018) and related maladaptive beliefs about others’ intentions, they may be particularly vulnerable to experiencing loneliness. In addition, a need for greater interpersonal distance has been repeatedly observed in people with psychotic disorders, when compared to healthy control samples (Deuš & Jokiá-Begić, 2006; Holt et al., 2015; Horowitz, Duff, & Stratton, 1964; Zapetis, Nasiriavanaki, Luther, & Holt, 2022), suggesting that an expectation of social threat or rejection, which can lead to or perpetuate experiences of loneliness, is present in many with these conditions.

In the current study, we employed an fMRI paradigm and analytic approach designed to elicit and account for the effects of these types of biases. During scanning, images of human faces were presented that appeared to move toward and away from participants, crossing participants’ personal space boundaries (Barbour et al., 2020; Holt et al., 2015, 2014; Nasiriavanaki et al., 2021; Vinke et al, 2025). Since an enlarged or more rigid personal space may represent a behavioral expression of the cognitive biases associated with loneliness (Layden et al., 2018; Saporta et al., 2021), we hypothesized that this fMRI paradigm engages some of the neural circuitry involved in the experience of loneliness. Following scanning, participants rated the trustworthiness of the face stimuli they had just viewed, and these ratings were incorporated in the fMRI analyses, based on the hypothesis that neural responses to the faces rated as least trustworthy are most relevant to the experience of loneliness. Thus, using this approach, we sought to identify neural responses associated with loneliness in individuals with and without psychotic disorders.

The findings of prior neuroimaging studies of loneliness have included associations between loneliness and responses or volumes of regions of the medial temporal lobe (Imai, Matsuoka, & Narumoto, 2022; Lieberz et al., 2022; Norbury, 2022; Saris et al., 2022; Tao et al., 2022; Zajner, Spreng, & Bzdok, 2021), basal ganglia (Cacioppo, Norris, Decety, Monteleone, & Nusbaum, 2009; Feng, Wang, Li, & Xu, 2019; Lieberz et al., 2021) and the hypothalamus (Wong et al., 2016). Also, several studies have identified associations between loneliness and the size (Düzel et al., 2019; Imai et al., 2022; Norbury, 2022; Tao et al., 2022) or function (Cacioppo et al., 2009; Lieberz et al., 2022; Saris et al., 2022; Zajner et al., 2021) of the hippocampus. In addition, studies conducted in rodents have found evidence for a central role of the amygdala (Lee, Chen, & Tye, 2021) and hypothalamus (Lee et al., 2021) in responses to social isolation, and studies in humans have also shown that the size (Zovetti, Rossetti, Perlini, Brambilla, & Bellani, 2021) and connectivity (Zovetti et al., 2021) of the amygdala are linked to social activity levels. Thus, in the current study, we tested the hypothesis that stronger associations between loneliness and responses of these previously implicated brain areas would be detected using an fMRI paradigm that appears to violate personal space boundaries, triggering a neural response to social threat. Additionally, because loneliness tends to correlate with social isolation, as well as depression and social anhedonia, we conducted secondary analyses that controlled for these variables, with the goal of isolating specific associations with loneliness. Finally, in a subset of individuals with longitudinal data, we tested if the responses of these brain areas predicted changes in loneliness over a one-year period.

## Methods

### Participants

#### Recruitment

Sixty healthy control subjects and 40 subjects with a diagnosis of a psychotic disorder (PD) were recruited and enrolled in this study (see Table 1 for participant characteristics). Of the 40 PD participants enrolled, three were later excluded due to having incomplete fMRI data (see Supplementary Methods for data quality assessment exclusion criteria). A research domain criteria (RDoC) approach (Cuthbert & Insel, 2013) was adopted for the design of this study, i.e. the PD category was defined broadly, including individuals with diagnoses of schizophrenia (*n* = 17), schizoaffective disorder (*n* = 9), and bipolar disorder with a history of psychosis (*n* = 11). See the Supplementary Methods for additional inclusion/exclusion criteria. Analyses were conducted both within each group and in the full sample, in order to fully test our dimensional hypotheses regarding the neural mechanisms of loneliness.

**Table 1.** Demographic and clinical characteristics of the sample

Measure	HC (*n* = 60) Mean (SD)	PD (*n* = 37) Mean (SD)	*p* – value	Test statistic
Gender (% female)	35.0	29.7	0.592	*X^2^* (1, *N* = 97) = 0.288
Age (yrs)	26.12 (5.53)	27.81(6.24)	0.166	t(95) = −1.395
Race^a^	12/6/35/7	8/5/24	0.971	t(95) = −0.037
Hispanic/Non-Hispanic	7/53	2/35	0.154	t(95) = −1.362
Mean parental education (yrs)	15.58 (3.33)	15.78 (2.86)	0.762	t(95) = −0.304
IQ^b^	109.92 (7.44)	106.71 (9.75)	0.072	t(95) = 1.822
Task performance^c^	80.7% (8.0%)	78.3%(10.2%)	0.204	t(95) = 1.280
Trustworthiness rating^d^	4.36 (0.17)	3.98 (0.72)	0.051	t(95) = −1.973
Personal space size^e^	79.40 (36.1)	107.25 (50.6)	0.002*	t(95) = −3.155
Loneliness at baseline^f^	29.94 (10.86)	39.61 (11.84)	< 0.001*	t(95) = −4.175
Loneliness at follow-up^g^	32.41 (12.02)	39.60 (10.42)	0.018*	t(60) = −2.435
Loneliness change^h^	1.76 (7.32)	0.56 (9.35)	0.575	t(60) = 0.564
Social activity levels^i^	18.48 (7.23)	15.58 (10.45)	0.002*	t(95) = 3.127
Depression^j^	2.94 (5.27)	7.84 (8.85)	< 0.001*	t(95) = −3.892
Social anhedonia^k^	7.89 (7.30)	10.65 (7.78)	0.089	t(95) = −1.719
Symptoms of schizophrenia^l^	–	67.14 (17.8)	–	–
Illness duration (yrs)	–	7.49 (6.03)	–	–
CPZ equivalents (mg)	–	253.93 (352.83)	–	–

Variables of interest listed in left-most column for Healthy Controls (HC) and Psychotic Disorders (PD) groups, with mean+/−standard deviation and *p* value for the between-group comparison (conducted using independent t-tests or chi-square tests), * *p* < .05. Variables include: gender (percentage of female), age (years), parental education (years)

a Race, the racial composition of the sample Asian/Black/White/No Response: 12 Asian, 6 Black, 35 White and 7 individuals with no response in the HC group; 8 Asian, 5 Black, 24 White in the PD group.

b Full-scale intelligence quotient (IQ; measured using the American National Adult Reading Test score).

c Performance on attention task during Looming paradigm.

d Trustworthiness ratings of face stimuli.

e Personal space size (measured using the Stop Distance Procedure) in centimeters.

f Loneliness score (measured using the UCLA loneliness scale) at baseline.

g Loneliness score (measured using the UCLA loneliness scale) 1 year post baseline in 35 HC and 31 PD participants.

h Loneliness change score (measured using the UCLA loneliness scale) from baseline to 1 year in 35 HC and 31 PD participants.

i Social activity levels (measured using the Social Network Index: number of social contacts).

j Depression (measured using the Beck Depression Inventory).

k Social anhedonia (measured using the Chapman Social Anhedonia Scale-Revised).

l Symptoms of schizophrenia (measured using the Positive and Negative Syndrome Scale: total score), illness duration (years), and chlorpromazine (CPZ) equivalents. The 37 PD subjects whose data were included in the analyses (subjects were excluded if found to have structural abnormalities of the brain (1 PD excluded) or very poor task performance during scanning (2 PD excluded; see Supplementary Materials for specific exclusion criteria)) had the following primary diagnoses: schizophrenia (*n* = 17), schizoaffective disorder (*n* = 9), and bipolar disorder with psychotic features (*n* = 11). The participants of the PD group were treated at the time of the study with the following antipsychotic medications: 43.2% (*n* = 16) aripiprazole, 13.5% (*n* = 5) clozapine, 13.5% (*n* = 5) olanzapine, 21.6% (*n* = 8) none, and the remaining 18.9% (*n* = 7) were being treated with one of the following medications: risperidone, quetiapine, lurasidone^m^, ziprasidone^m^, perphenazine, haloperidol, cariprazine, or paliperidone palminate (^m^ same individual).

Participants were recruited via advertisements in online community forums and postings on research portals (Rally; Clinical trials https://rally.massgeneralbrigham.org/). PD participants were recruited either through online advertisements or the MGH Psychosis Clinical and Research Program (PCRP). DSM-V diagnoses of all participants were evaluated by trained research staff using the Mini International Neuropsychiatric Interview (Sheehan et al., 1998).

In accordance with the Declaration of Helsinki, written informed consent was obtained from all participants before the beginning of the study. All study procedures were approved by the Massachusetts General Brigham Institutional Review Board.

#### Clinical assessment and loneliness and social isolation measures

In all participants, loneliness was assessed using the UCLA Loneliness Scale (Russell, 1996), which is a well-validated, self-report measure of loneliness that has been used in many populations, including psychotic disorder samples (Badcock et al., 2015; Ludwig et al., 2020). Social activity levels were measured using the Social Network Index, which assesses the number of social contacts that the participant had during the previous 2 weeks (Cohen, Doyle, Skoner, Rabin, & Gwaltney, 1997). Also, depression (Beck, Ward, Mendelson, Mock, & Erbaugh, 1961), social anhedonia (Chapman, Chapman, & Raulin, 1976) and the symptoms of schizophrenia (in the PD group only) (Kay, Fiszbein, & Opler, 1987) were measured using well-validated instruments. In addition, the identical assessments were also administered 1 year following the baseline time point (collected in the 35 HC and 31 PD participants who were willing to participate in the follow-up assessment).

#### Functional MRI task (the ‘Looming’ paradigm)

At baseline only, participants underwent an MRI scan session, which included a well-validated fMRI paradigm (Barbour et al., 2020; Holt et al., 2015, 2014; Nasiriavanaki et al., 2021; Vinke et al., 2025) during which participants view images of human faces (eight female and eight male – appearing digital face images, each with a neutral facial expression, half with eyes open and half with eyes closed, created using https://facegen.com), which either increase in size (appear to be approaching the participant) or decrease in size (appear to be withdrawing from the participant) over the course of a 16-second trial. Each functional run included a total of 16 trials, with 16-second blank (neutral gray) fixation blocks presented at the beginning and end of each functional run. Face gender and looming trial type (approach, withdrawal) were randomized and counter-balanced within each functional run.

#### Attentional task during scanning

While maintaining central fixation throughout each functional run, participants were instructed to covertly attend to other areas of the screen and report whenever they detected the appearance of a dot at a random location on the screen. During each 16-second looming trial, a dot appeared 3 times, with the duration between subsequent dot presentations randomly varying between 3.5 s and 4.5 s. Additionally, the duration of each dot presentation varied randomly between 350 ms and 1,500 ms, with the dot size scaled with eccentricity (i.e. with a larger diameter when presented further from central fixation). Participants responded using their right index finger to press a key on a response box.

There was no significant difference between the HC and PD groups in performance on this attentional task (*p* = .204).

#### Face ratings and personal space measurement

Following the scan session, each participant was shown the face stimuli they had viewed during the scanning, one at a time, in a randomized order. Each of the 16 faces was shown four times. During the viewing of each face, participants rated (self-paced) the trustworthiness of the face (among other ratings) on a scale of 0–10 (see Supplementary Figure S1). In addition, personal space size was measured on the same day as the scan session using the Stop Distance Procedure (SDP), which is a validated, reliable procedure for measuring personal space preferences (Hayduk, 1983). During the SDP, a confederate who is unknown to the subject stands 3 meters away from (and facing) the subject, and then slowly walks directly toward the subject, while maintaining a neutral facial expression and eye contact throughout the procedure. The subject stops the confederate at two points: first when they begin to feel ‘slightly uncomfortable’, that is, when their personal space boundary has just been reached (‘the distance at which you would normally have a conversation with a person you have just met’) and second when they feel ‘very uncomfortable’, that is, when their personal space has been entered.

### MRI data analysis

All MRI data analyses were conducted using FreeSurfer v7.4.1. See Supplementary Methods for the structural and functional MRI data preprocessing procedures and the data quality assurance methods used.

#### Regions-of-interest (ROI) definition

The Looming paradigm consistently engages a network of cortical and subcortical regions (Barbour et al., 2020; Holt et al., 2015, 2014; Nasiriavanaki et al., 2021; Vinke et al, 2025), which were defined in this study using (1) previously collected data acquired using this paradigm or (2) anatomical criteria. An independent fMRI dataset collected with the Looming paradigm (in a non-clinical sample of young adults (*n* = 130)) was used to independently define the cortical ROIs (Nasiriavanaki et al., 2021). These *a priori* cortical ROIs included bilateral parietal and ventral premotor cortical regions (see Supplementary Methods). Activation in these cortical ROIs was assessed as a positive control to establish that the task engaged the expected cortical regions (Barbour et al., 2020; Holt et al., 2015, 2014; Nasiriavanaki et al., 2021; Vinke et al, 2025). The hypothesis-testing analyses focused on *a priori* subcortical brain regions that have been implicated in prior studies of the neural correlates of loneliness (see Introduction): the medial temporal lobe (the amygdala and hippocampus) (Cacioppo et al., 2009; Düzel et al., 2019; Feng et al., 2019; Imai et al., 2022; Lieberz et al., 2022; Norbury, 2022; Saris et al., 2022; Tao et al., 2022; Wong et al., 2019; Zajner et al., 2021), the basal ganglia (the caudate nucleus, putamen, nucleus accumbens, pallidum, and thalamus) and the basal forebrain, which includes the hypothalamus (Cacioppo et al., 2009; Feng et al., 2019; Lieberz et al., 2021, 2022; Wong et al., 2016). These eight subcortical ROIs were defined using the automatic subcortical segmentation algorithm of Freesurfer (https://surfer.nmr.mgh.harvard.edu/).

#### Functional MRI data analyses

The first-level analysis used a univariate general linear model (GLM), fit to the event-related BOLD time-series data, for all runs passing quality assurance steps. The GLM included a canonical SPM hemodynamic response function, head motion (six parameters) and scanner drift variables as nuisance regressors and excluded any time-points identified as outliers (see Supplementary Methods for additional details). The GLM contrast effect size (CES) maps were generated when contrasting responses of all approaching face trials versus all withdrawing face trials (i.e. the Approach vs. Withdrawal contrast, referred to as ‘looming’), using each participant’s individual trustworthy rating of each face as run-specific parametric regressors (e.g. a model comprised of the Approach vs. Withdrawal (looming) contrast together with the trustworthy ratings for each subject) (Wood, Nuerk, Sturm, & Willmes, 2008).

Both cortical and subcortical group-level analyses were performed to identify regions where the group-wise CES maps were significantly different from zero; significance maps were thresholded at *p* < 0.05, and false discovery rate (FDR) permutation-testing (cluster-wise *p*-value <0.05) was applied for whole-brain correction, for the cortical surface and subcortical space separately. Between-group differences were initially examined in the *a priori* ROIs using whole-brain voxel-wise comparisons conducted at a liberal *p* < 0.05 uncorrected threshold in order to facilitate comparisons to prior studies. These analyses were followed by analyses that (1) controlled for potential confounds and (2) used a whole-brain *p* < 0.05 cluster-wise FDR-corrected threshold.

#### Whole brain regressions using loneliness as a regressor

Regression analyses were conducted using a GLM voxel-wise technique utilizing the CES maps (approach vs. withdrawal contrast X trustworthy regression coefficient), to measure the relationship between Looming activation and loneliness in the HC, PD, and full samples. Additional second-level GLM regression analyses, conducted in the full sample only, included (1) measures of potential confounds as covariates and (2) change in loneliness from baseline to the 1-year follow-up time point.

## Results

### Loneliness, social activity levels, and clinical measures

Levels of loneliness were significantly greater on average in the PD group than in the HC group (*t*(97) = −4.175, *p* < 0.001; Table 1), and mean social activity levels were significantly lower in the PD, compared to the HC, group (*t*(97) = 3.175, *p* = 0.002). At the 1-year follow-up timepoint, loneliness remained significantly greater in the PD, compared to the HC, group (*t*(60) = −2.435, *p* = 0.018), but the change in loneliness from baseline to 1 year was not significantly different between the two groups (*t*(60) = 0.560, *p* = 0.575) (Table 1).

### Neural responses to looming faces

#### Within-group effects

**Figure 1.**
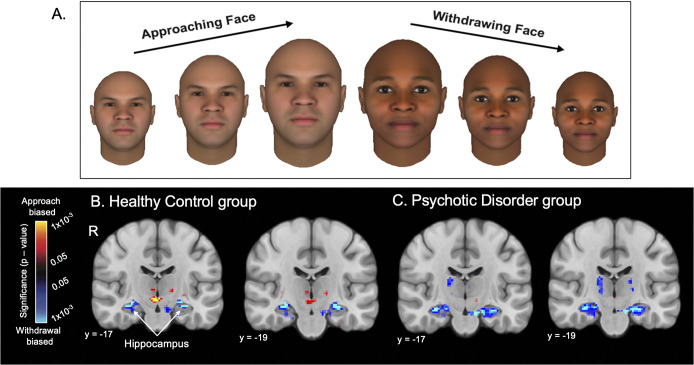
The Looming task stimuli and looming activation within the Healthy Control and Psychotic Disorder groups. (a) Examples of the Looming task face stimuli are shown. During a trial, one unique face stimulus either increases (Approach condition) or decreases (Withdrawal condition) in size over the course of a 16 second block. The face displayed on the left (approaching, three images) was rated the least trustworthy face on average, while the face shown on the right (withdrawing, three images) was rated the most trustworthy face on average, across the full sample (*n* = 97). (b) The functional contrast of Approach > Withdrawal was computed to assess neural responses to the Looming task. Subcortical voxel-wise activation (Approach > Withdrawal) maps are shown for the Healthy Control (*n* = 60) and Psychotic Disorder (*n* = 37) groups, with a significance map (display threshold: *p* < 0.05, uncorrected) overlaid on the MNI common space brain template, showing significant Withdrawal > Approach responses of the right and left hippocampus in two coronal slices (y = −17 and y = −19) in the two groups. Approach biased, Approach > Withdrawal; R, right hemisphere; Withdrawal biased, Withdrawal > Approach.

There were significant ‘looming’ (Approach > Withdrawal) effects in all cortical ROIs in both groups and the full sample (Supplementary Table S2 and Figure S2). In subcortical areas, in the HC group, there were significant looming effects within the bilateral basal forebrain and right thalamus, left hippocampus, left caudate, and left putamen (Supplementary Table S2 and Figure 1), whereas in the PD group, significant looming effects were found in the left basal forebrain, bilateral thalamus, bilateral hippocampus, and left amygdala (Supplementary Table S2 and Figure 1).

#### Between-group comparisons

At a liberal threshold (*p* < .05 uncorrected), some significant differences between the two groups were evident in the bilateral basal forebrain, bilateral hippocampus, left amygdala, bilateral caudate, and left putamen (Supplementary Table S3 and Figure S3), with greater responses Approach > Withdrawal activation in the HC compared to the PD group – primarily due to elevated Withdrawal > Approach activation in the PD group. These differences were not significant, however, after controlling for between-group differences in loneliness or social activity levels (Supplementary Figure S3). Also, there were no significant differences between the two groups in activation in any of the ROIs at a *p* < 0.05 cluster-wise, FDR-corrected threshold.

### Neural correlates of loneliness

#### In the separate HC and PD groups

**Figure 2.**
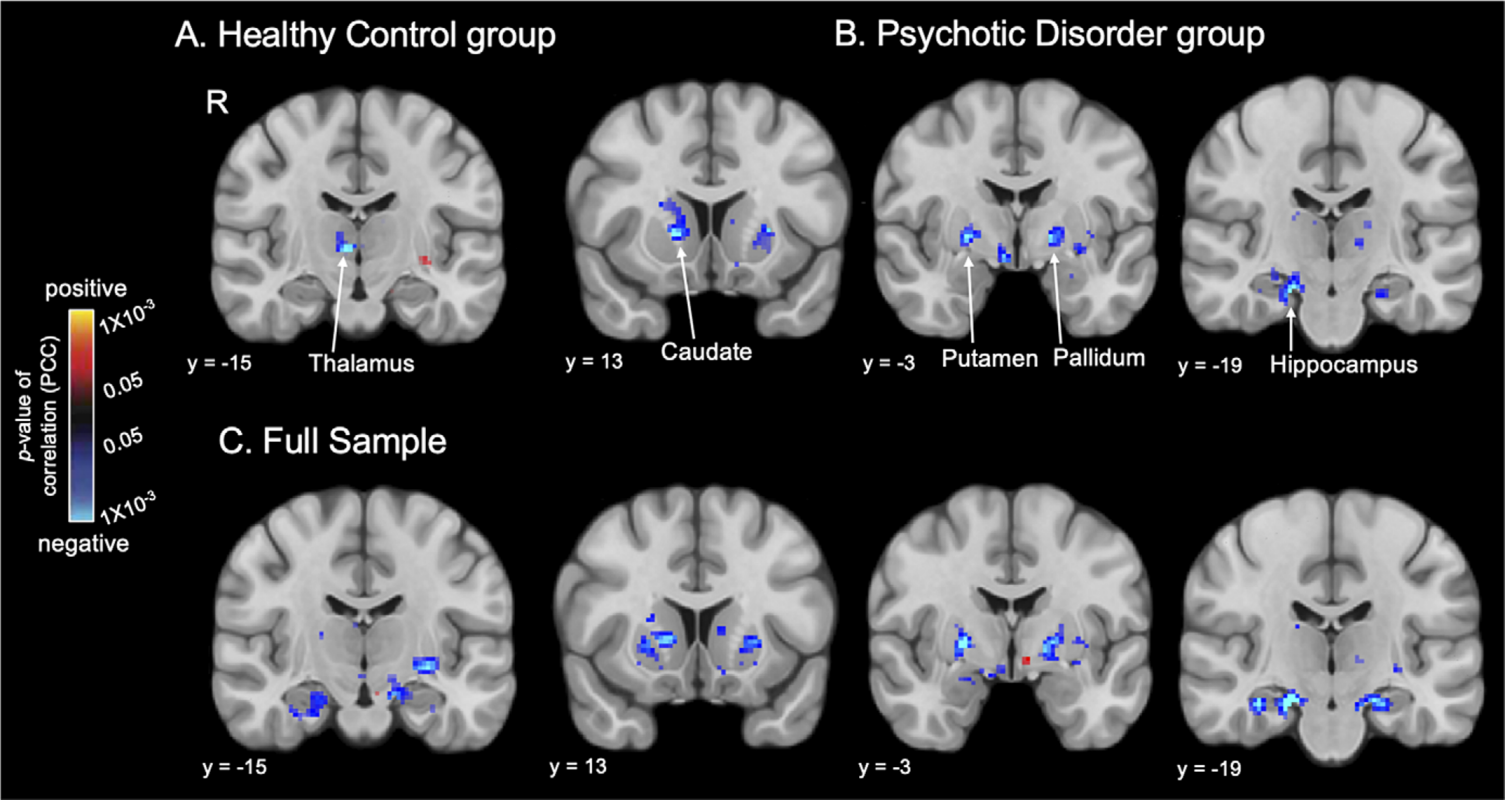
Associations between looming responses of subcortical areas and loneliness. Maps of the results of whole brain regressions show the significant associations between self-reported loneliness and looming (Approach > Withdrawal) activation in subcortical areas, overlaid on a MNI common space brain template (display threshold: *p* < 0.05, uncorrected) in the Healthy Control (A, *n* = 60), Psychotic Disorder (B, *n* = 37) groups and full sample (C, *n* = 97). Warm colors (red, yellow) indicate voxels showing significant positive correlations, and cool colors (blue) indicate voxels showing significant negative correlations between Approach > Withdrawal activation and loneliness. Thus, voxels labeled blue also represent those showing significant positive correlations between loneliness and Withdrawal > Approach activation. PCC, partial correlation coefficient; R, right hemisphere.

**Figure 3.**
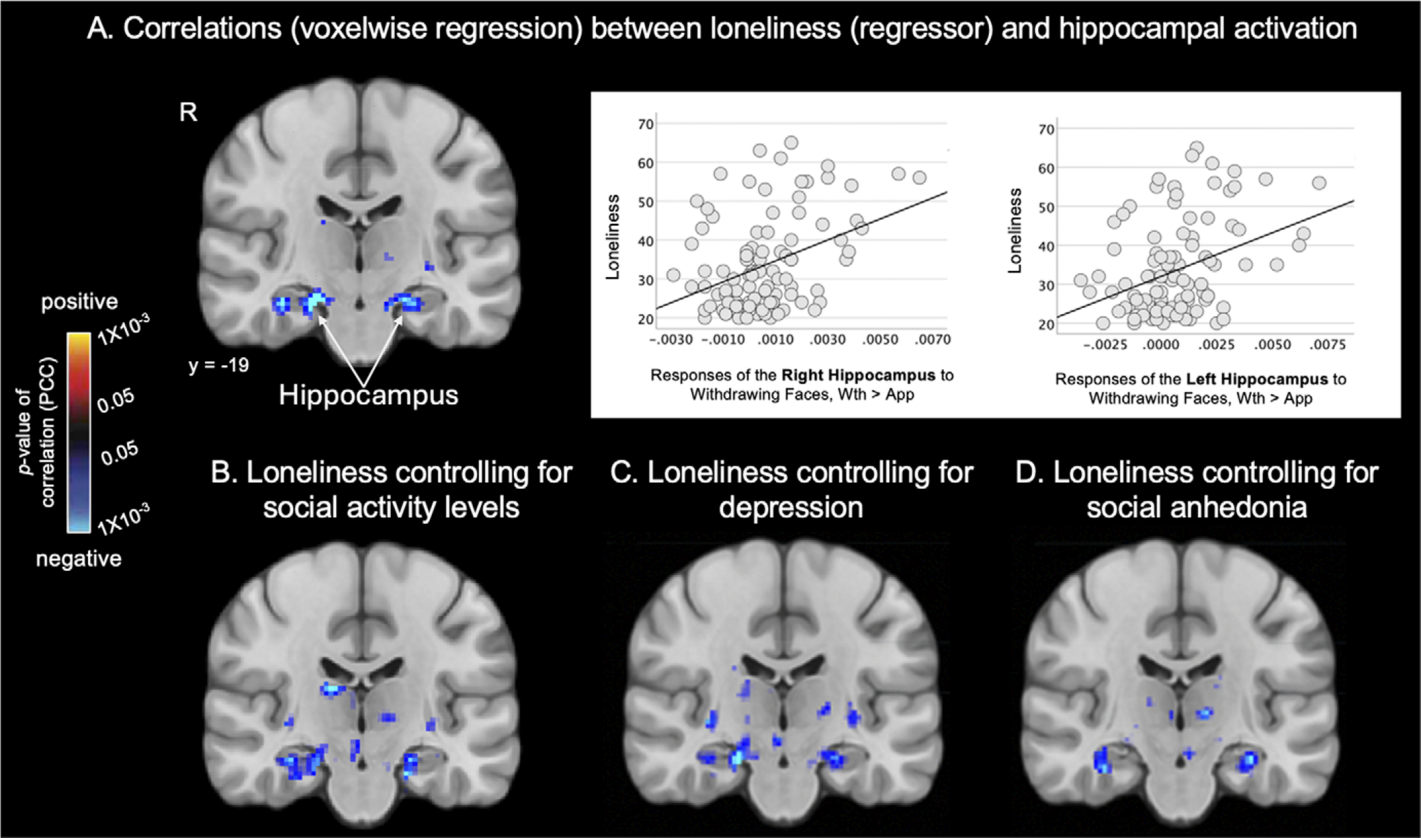
Loneliness is associated with looming responses of the hippocampus, even after controlling for potential confounds. Maps of the results of whole brain regressions conducted in the full sample (*n* = 97) show the significant associations between loneliness and looming (Approach > Withdrawal) activation in the hippocampus. Subcortical significance maps (display threshold: *p* < 0.05, uncorrected) resulting from the voxel-wise regression analysis testing for associations between loneliness and looming activation without covariates (a) and when controlling for social activity level (b), depression (c) or social anhedonia (d) are shown. Plots in (a) demonstrate the significant association between Withdrawing > Approaching faces and loneliness in the whole sample. Warm colors (red, yellow) indicate voxels showing significant positive correlations (none are shown here), and cool colors (blue) indicate voxels showing significant negative correlations between Approach > Withdrawal activation and loneliness. Thus, voxels labeled blue also represent those showing significant positive correlations between loneliness and Withdrawal > Approach activation. App, approach; PCC, partial correlation coefficient; R, right hemisphere; Wth, withdrawal.

Whole-brain regressions testing for an association between loneliness and looming-related activation of *a priori* subcortical areas were then conducted (*p* < 0.05, FDR-corrected). In the HC group, a significant cluster was observed within the right thalamus (peak *Z* = −3.830, *p* = 1 × 10^−4^); this association arose from a negative correlation between Approach > Withdrawal responses of the right thalamus and loneliness (Table 2; Figure 2). In the PD group, a similar (and stronger) pattern of results was found; levels of loneliness were significantly negatively correlated with Approach > Withdrawal activation, most strongly in the right hippocampus (peak *Z* = −4.345, *p* = 1×10^−5^), as well as in the right putamen, right caudate nucleus, and left pallidum (Table 2; Figure 2). This pattern of results can also be represented as positive correlations between loneliness and Withdrawal > Approach responses (Figure 3A). See Supplementary Table S4 and Figure S4 for additional results.

**Table 2.** Locations of clusters (surviving whole brain correction) showing negative correlations between responses to looming (Approach > Withdrawal) faces and loneliness in the subcortical regions-of-interest in the Healthy Control (HC), Psychotic Disorder (PD) and full samples

Region	Hemisphere	Cluster size (mm^3^)	Talaraich Coordinates (x,y,z) of peak	Peak Z-statistic	Significance Level (*p*-value) of peak voxel	Cohen’s d
a) HC group (*n* = 60)						
Thalamus	right	56	6, −15, 1	−3.830	1×10^−4^	−0.494
b) PD group (*n* = 37)						
Hippocampus	right	32	28, −35, −3	−4.345	1×10^−5^	−0.714
Caudate	right	16	16, 13, 1	−3.323	9×10^−4^	−0.546
Putamen	right	8	18, 7, −15	−3.159	2×10^−3^	−0.519
Pallidum	Left	136	−20, 1, −1	−3.068	2×10^−3^	−0.504
c) Full sample (*n* = 97)						
Hippocampus	Left	24	−24, −23, −17	−3.457	5×10^−4^	−0.351
Hippocampus	right	8	34, −19, −19	−4.237	2×10^−5^	−0.430
Putamen	Left	224	−20, 3, −5	−4.237	2×10^−5^	−0.430
Putamen	right	8	22, 5, −11	−3.175	2×10^−3^	−0.322
Pallidum	right	152	20, 3, −3	−3.995	6×10^−5^	−0.406
Ventral DC	Left	8	−14, −17, −19	−3.081	2×10^−3^	−0.313
Ventral DC	right	72	18, −19, −17	−3.194	1×10^−3^	−0.324

A whole-brain permutations cluster-wise FDR corrected *p* < .05 was used to determine significance using Freesurfer’s mri_glmfit. Subcortical areas examined included the medial temporal lobe (amygdala and hippocampus), basal ganglia (caudate, putamen, nucleus accumbens, pallidum, and thalamus), and basal forebrain (labeled as the ventral diencephalon (Ventral DC) in Freesurfer—this ROI includes, but is not limited to, the hypothalamus).

*In the full sample.* Given that there were no significant differences between the HC and PD groups in activation at a whole-brain-corrected statistical threshold, the above regression analyses were repeated in the full sample (*n* = 97) to gain additional power. In the full sample, loneliness was most strongly correlated with looming-related activation of the right hippocampus (peak *Z* = −4.237, *p* = 2×10^−5^). Similar effects were also observed in the left hippocampus, bilateral basal forebrain, right pallidum, and bilateral putamen (Table 2; Figure 2).

#### Controlling for potential confounds

Because loneliness was correlated to varying degrees with social isolation (i.e. low social activity levels), depression, and social anhedonia (Supplementary Table S1), the regression analysis testing for associations between loneliness and looming-related activation was repeated in the full sample using these variables as covariates. The associations between loneliness and responses of the right and left hippocampus were the only findings that remained significant when controlling for each of these variables (Supplementary Table S5 and Figure 3).

#### Neural predictors of change in loneliness over 1 year

**Figure 4.**
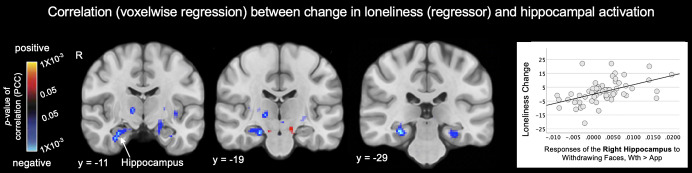
Responses of the hippocampus at baseline predict change in loneliness one year later. Maps of the results of whole brain voxel-wise regressions (display threshold: *p* < 0.05, uncorrected) conducted in a subset of the full sample (*n* = 66) show the significant associations between Approach > Withdrawal activation of the right hippocampus (*p* < 0.05, FDR-corrected) at baseline and changes in loneliness between baseline and the one-year follow-up time point. The scatter plot illustrates this association, displaying the positive correlation between the responses of the right hippocampus to Withdrawing > Approaching faces at baseline and changes in loneliness over one year (larger responses were associated with increases in loneliness). In A, warm colors (red, yellow) indicate voxels showing significant positive correlations, and cool colors (blue) indicate voxels showing significant negative correlations between Approach > Withdrawal activation and change (One Year > Baseline) in loneliness. Thus, voxels labeled blue also represent those showing significant positive correlations between an increase in loneliness over one year and Withdrawal > Approach activation at baseline. App, approach; PCC, partial correlation coefficient; R, right hemisphere; Wth, withdrawal.

A longitudinal regression analysis in the subsample with both baseline and 1-year follow-up data (35 HC and 31 PD participants) revealed that looming-related activation of the right hippocampus, but no other brain region, predicted greater loneliness at 1-year follow-up (peak *Z* = −3.223, *p* = 2 × 10^−4^; Figure 4).

## Discussion

### Summary of main findings

The goal of this study was to identify brain responses specifically linked to loneliness in healthy individuals and those diagnosed with a psychotic disorder. In both the psychotic disorder group and the full sample (and at a lower threshold in the healthy control group), loneliness was correlated with responses of the hippocampus to withdrawing (vs. approaching) face stimuli. In addition to the hippocampus, responses of basal ganglia regions (the right caudate and putamen and left pallidum in the psychotic disorder group, the right thalamus in the healthy control group) to withdrawing versus approaching faces showed a similar relationship to loneliness. Moreover, only the association between loneliness and responses of the hippocampus remained significant in the full sample after controlling for potential confounding factors (social activity levels, depression, social anhedonia). The central role of the hippocampus in the experience of loneliness was further highlighted by the finding that hippocampal responses also predicted increases in loneliness 1 year later in a subset of the full sample.

### Prior evidence for a role of the hippocampus in loneliness

The hippocampus has been consistently implicated in prior neuroimaging studies of loneliness conducted in a number of different populations (Lam et al., 2021). Several studies, including two conducted in large samples (*N* = ~2,000 (Tao et al., 2022) and ~ 40,000 (Norbury, 2022)), have detected significant negative correlations between loneliness and hippocampal volumes (Düzel et al., 2019; Imai et al., 2022; Norbury, 2022; Tao et al., 2022; Wong et al., 2019). Also, loneliness has been associated with reductions in the integrity of white matter fibers both intrinsic to and originating from the hippocampus (Spreng et al., 2020; Wong et al., 2016).

In addition to these structural findings, loneliness has also been linked to altered functional connectivity and task-elicited responses of the hippocampus (Lieberz et al., 2021, 2022; Saris et al., 2022; Wong et al., 2019; Zajner et al., 2021). For example, altered functional connectivity of the CA1 subfield and molecular layer of the hippocampus with regions of the default network has been demonstrated in individuals endorsing loneliness in the UK Biobank sample (Zajner et al., 2021). In addition, diminished hippocampal-nucleus accumbens connectivity during negative social feedback was observed in a group of healthy individuals with high (vs. low) levels of loneliness (Lieberz et al., 2022). Taken together, mounting evidence suggests that changes in hippocampal structure and function are linked with loneliness.

Despite the consistency of these findings across distinct neuroimaging methods and experimental paradigms, the neurobiological mechanisms underlying these associations remain poorly understood. Given that loneliness has been linked to increases in cortisol and peripheral physiological measures of stress in humans (Doane & Adam, 2010), a reduction in hippocampal volume in lonely individuals (Düzel et al., 2019; Imai et al., 2022; Norbury, 2022; Tao et al., 2022; Wong et al., 2019) may reflect changes in hippocampal morphology and neurogenesis (Lee et al., 2021) that are similar to those previously detected in animal models of the effects of chronic stress (McEwen, Nasca, & Gray, 2016). Future longitudinal studies can investigate whether loneliness-related stress responses lead to changes in hippocampal structure and function in humans and determine whether loneliness precedes or follows such changes.

The possibility that the hippocampus may play a role in encoding and/or retrieving the cognitive biases that increase susceptibility to loneliness (i.e. sensitivity to social threat or rejection), or the conscious experience of loneliness itself, is consistent with evidence that the hippocampus is involved in generating numerous types of cognitive maps, including ‘social maps’ which involves tracking and comparing levels of affiliation between the self and others (Montagrin, Saiote, & Schiller, 2018). Studies which attempt to manipulate aspects of this social mapping could test whether changes in these hippocampal-dependent processes lead to subsequent changes in levels of loneliness.

### The basal ganglia in loneliness

The current study also found that responses of basal ganglia regions, including the caudate nucleus, putamen, and thalamus, to withdrawing (vs. approaching) faces correlated with loneliness. However, these effects did not remain significant after controlling for potentially confounding factors, such as social activity levels, social anhedonia, and depression. Thus, one possible interpretation of this pattern of results is that the basal ganglia play a role in experiences or symptoms that often co-occur with loneliness. Consistent with this interpretation, one prior study showed that decreased gray matter volumes of the pallidum, putamen, and caudate nucleus were significantly correlated with loneliness in a group of older adults with a history of multiple depressive episodes (Sin et al., 2018). Given the known role of the basal ganglia in reward-related processes (Vitale & Smith, 2022), it is possible that basal ganglia circuitry is involved in triggering responses (including changes in mood and actions) to deficits in social connection and to corrections of such deficits (Fulford & Holt, 2023; C. R. Lee et al., 2021).

### Associations between loneliness and hippocampal responses in psychotic disorders

Many prior neuroimaging studies employing a case–control design and a hypothesis-driven, ROI approach have detected significant reductions (on average) in hippocampal volume or abnormalities in hippocampal responses in groups of individuals diagnosed with schizophrenia, when compared with healthy control subjects (Heckers & Konradi, 2010; Knight et al, 2022). In the current study, when a liberal statistical threshold was used (comparable to many prior hypothesis-driven, ROI focused studies), significant between-group differences in activation of the hippocampus and other regions were found, with significantly greater responses of those regions to approaching versus withdrawing faces in the healthy control group compared to the psychotic disorder group. These between-group differences in the hippocampus and other regions were driven by the greater responses to withdrawing (compared to approaching) face stimuli in the psychotic disorder group; since these responses were correlated with loneliness, the high levels of loneliness in the psychotic disorder group may have contributed to these findings. This possibility was supported by subsequent analyses that included loneliness as a covariate, which found no significant between-group differences in activation. Thus, overall, this pattern of results suggests that the effects on the brain of chronic loneliness, experienced by ~80% of people with schizophrenia (Stain et al., 2012), may be an important variable to consider when investigating neural changes associated with schizophrenia and other serious mental illnesses.

It has been proposed that social isolation and loneliness may increase susceptibility to developing psychosis, by creating conditions (a ‘social deafferentation’) that foster the emergence of potentially compensatory phenomena such as hearing people talking, which may provide a type of simulation of social activity in response to loneliness (Hoffman, 2008). In light of the evidence for a role of the hippocampus in loneliness, and prior evidence for abnormalities in hippocampal structure and function (Allen et al., 2016; Dean et al., 2016; Ho et al., 2017; Sasabayashi et al., 2021; Schobel et al., 2009) and in social disconnection-related changes in hippocampal functional connectivity (Aberizk et al., 2025) in psychosis risk states, one hypothesis that could be tested in future studies is that changes in hippocampal function linked to loneliness precede, and increase the likelihood of, psychosis onset. However, the inconsistent evidence for a predictive relationship between hippocampal abnormalities in psychosis risk states and later onset of psychotic disorders (Allen et al., 2016; Hinney, Walter, Aghlmandi, Andreou, & Borgwardt, 2021; Provenzano et al., 2020; Schobel et al., 2009; S. J. Wood et al., 2010) must also be considered when evaluating this model.

### Longitudinal findings

A longitudinal analysis in a subset of the full sample revealed that the hippocampal responses to withdrawing (vs. approaching) faces at baseline also predicted worsening loneliness over time. This finding suggests that this hippocampal response may represent a marker of the neurocognitive processes that underlie loneliness. Additional studies will be necessary to further confirm this finding and identify the processes that rely upon the hippocampus (such as autobiographical memory (Sheldon, Fenerci, & Gurguryan, 2019) or mapping of social affiliation (Tavares et al., 2015)) that may play a key role in the experience of loneliness.

### Limitations

This study has several limitations to consider when interpreting its results. First, direct causal associations cannot be inferred regarding the relationships observed between neural responses and loneliness. Second, a self-report scale was used to measure loneliness in this study; future studies could also include *in vivo* measures, such as ecological momentary assessments (Mote & Fulford, 2020) or wearable devices that measure aspects of social activity, to capture different determinants and expressions of loneliness. However, the self-report measure used in the current study, the UCLA loneliness scale, has been well-validated across many populations and found to correlate with momentary measures of loneliness (Alsubheen, Oliveira, Habash, Goldstein, & Brooks, 2023; van Winkel et al., 2017), suggesting that it roughly captures the day-to-day conscious experience of feeling lonely for many. Also, although previous research has identified relationships between loneliness and gender, race, ethnicity, socioeconomic status, and age (Kannan & Veazie, 2023; McCutcheon et al., 2018; Schmitt & Kurdek, 1985), we did not find any such effects in our data, likely due to limitations in power.

### Future directions

Follow-up studies can investigate the psychological and biological processes and mediators (e.g. stress-related, inflammatory, hormonal) that may account for the associations observed in this study. An understanding of such mechanisms may lead to the development of interventions that specifically target these processes, potentially improving the physical health and overall quality of life of lonely individuals.

For example, future research can investigate whether the hippocampus and its network (and/or other brain regions) play a specific role in measuring discrepancies between expected and actual (perceived) levels of social connection (in line with the ‘social homeostasis’ model of loneliness (Matthews & Tye, 2019)), as well as the subjective responses (feelings of loneliness) to such discrepancies. Studies that aim to modify this evaluation process, potentially by reducing the ‘expected’ or increasing the ‘actual’ level of social connection experienced by an individual, potentially via cognitive behavioral or mindfulness approaches previously found to reduce loneliness in some populations, could then target the neural systems involved (Holt, 2025). Emerging lines of evidence show that mindfulness-based interventions lead to reductions in loneliness (Detore, Burke, Nyer, & Holt, 2024; Lindsay, Young, Brown, Smyth, & David Creswell, 2019; Saini, Haseeb, Taghi-Zada, & Ng, 2021; Teoh, Letchumanan, & Lee, 2021) and to decreases in stress and inflammation (Lee, Tsai, Yu, & Chan, 2025; Pascoe, Thompson, Jenkins, & Ski, 2017), as well as changes in the hippocampus, among other brain regions (Fox et al., 2014; Gotink, Meijboom, Vernooij, Smits, & Hunink, 2016; Hölzel et al., 2011; Luders, Toga, Lepore, & Gaser, 2009), supporting one testable model of loneliness. A better understanding of the sequence of neural events that perpetuate states of loneliness may also contribute to destigmatizing this common experience, which is an initially adaptive response that can become detrimental over time in an increasingly socially fragmented world.

## Supporting information

10.1017/S003329172510250X.sm001 McKenna et al. supplementary material McKenna et al. supplementary material

## Supplementary material

The supplementary material for this article can be found at http://doi.org/10.1017/S003329172510250X.
